# Extraction, Structural Characterization, and Potential Antioxidant Activity of the Polysaccharides from Four Seaweeds

**DOI:** 10.3390/ijms17121988

**Published:** 2016-11-28

**Authors:** Jinzhe He, Yaoyang Xu, Hongbo Chen, Peilong Sun

**Affiliations:** 1 Department of Food Science and Engineering, Zhejiang University of Technology, Hangzhou 310014, Zhejiang, China; hejzgd@163.com (J.H.); xuyaoyang1030@126.com (Y.X.); 2 Zhejing Fangyan Test Group Co., Ltd., Hangzhou 310018, Zhejiang, China; see271@126.com

**Keywords:** seaweed, polysaccharide, extraction, molecular weight (*M*_W_), composition, antioxidant activities

## Abstract

Four seaweed polysaccharides were extracted from *Sarcodia ceylonensis*, *Ulva lactuca* L., *Gracilaria lemaneiformis*, and *Durvillaea antarctica*, respectively, by microwave-assisted extraction. The effect of three significant variables (extraction time, extraction temperature, and the ratio of water to raw material) on the process for extracting polysaccharides was investigated, along with the optimization of the extraction using the response surface method (RSM) with a Box–Behnken design. The polysaccharide structure, monosaccharide composition, degree of sulfation, and molecular weight (*M*_W_) distribution were analyzed by infrared (IR) spectrometry, gas chromatography (GC), and high-performance gel permeation chromatography (HPGPC). IR spectrometry showed that *Sarcodia ceylonensis* polysaccharide (SCP), *Ulva lactuca* L. polysaccharide (ULLP), and *Durvillaea antarctica* polysaccharide (DAP) were all sulfated polysaccharides and, except *Gracilaria lemaneiformis* polysaccharide (GLP), all belong to β-pyranosidic polysaccharides. The average molecular weight (*M*_W_) of SCP, ULLP, GLP, and DAP was 466, 404, 591, and 482 kDa, respectively. The quantitative and comparative results with external standards indicated that the main monosaccharide in SCP and ULLP was mannose; and GLP and DAP were mainly composed of galactose and glucose, respectively. Then the in vitro antioxidant activity of all of the polysaccharides was evaluated using different assays—2,2–azino –bis (3-ethylbenzthiazoline-6- sulfonate) (ABTS), hydroxyl radical, nitrite scavenging capacity, and reducing power—and the relationship between their antioxidant activity and chemical characteristics were also examined. ULLP presented the highest ABTS radical scavenging activity; ULLP, SCP and DAP also showed a strong effect on the ABTS radical scavenging activity. SCP and ULLP exhibited excellent hydroxyl radical scavenging activities, about 83.33% ± 2.31% and 80.07% ± 2.17%, respectively, at 4 mg/mL. The reducing power of DAP was relatively more pronounced than that of the three other polysaccharides. However, the nitrite scavenging activities of the four seaweed polysaccharides were weaker than other antioxidant activity (ABTS), hydroxyl radical scavenging capacity, and reducing power. In addition, GLP exhibited lower activities than the other three samples in all of the tests for the antioxidant activity.

## 1. Introduction

Seaweeds belong to the lower cryptogams and are mainly composed of green algae, red algae, and brown algae; they are rich in protein, a variety of vitamins, minerals, and fibers, and have been widely used in the food and pharmaceutical industries [1,2,3]. Nowadays, polysaccharides from seaweed are becoming more and more important in biochemical and medical areas [4,5]. The major functional compounds of seaweeds are the various polysaccharides that are abundantly present in the structural features of their cells. Particularly, more evidence has been reported that many bioactive polysaccharides not only possess antiviral, anti-tumor, immunomodulation, and anti-inflammatory and antioxidant properties, but also relatively low toxicity and high bioactivities [6,7].

In recent years, several reports have revealed that seaweed polysaccharides exhibit strong free-radical scavenging activity and can be used as antioxidants for the prevention of oxidative damage in living organisms [8], and significant evidence has indicated that the biological activities of polysaccharides depend on their structural features, such as the degree of sulfation and distribution pattern of sulfate, type of glycosidic linkages, branch structures, molecular weight, and monosaccharide composition [9,10]. In addition, some β-(1→3), (1→6) glycosidic linkages are supposed to play important roles in enhancing the antitumor and the immunomodulatory effects [11,12,13]. However, little attention has been given to the comparative investigation of the chemical composition, degree of sulfation and respective distribution pattern, antioxidant potential, glycosidic linkages, and molecular weight (*M*_W_) of the polysaccharides from different seaweeds. Therefore, evaluation and comparison of the polysaccharides of different kinds of algae, considering their chemical composition and bioactivities, were particularly necessary to broaden their applications in pharmaceutical industries and functional foods.

The objectives of this work were to study microwave-assisted extraction (MAE) of polysaccharides, and compare the structure, monosaccharide composition, *M*_W_, and antioxidant activity of the polysaccharides from four different seaweeds, including one green alga *Ulva lactuca* L., two red algae *Gracilaria lemaneiformis* and *Sarcodia ceylonensis*, and one brown alga *Durvillaea antarctica*. These four seaweeds are commonly found off the coast of China. These results might be useful for further biochemical studies in the future.

Microwave-assisted extraction (MAE) was used and optimized with the aim at efficiently extracting the crude polysaccharides from the algal samples. The effect of three significant variables (extraction time, extraction temperature, and the ratio of water to raw material) on the process for extracting polysaccharides were investigated, along with the optimization of the extraction using response surface method (RSM) with a Box–Behnken design (BBD) [14]. The chemical and monosaccharide composition of polysaccharides were determined. Then the in vitro antioxidant activity of all the polysaccharides was evaluated using different assays—2,2-azino -bis(3-ethylbenzthiazoline-6-sulfonate (ABTS), hydroxyl radical, nitrite scavenging capacity and reducing power—and the relationship between their antioxidant activity and chemical characteristics were also examined.

## 2. Results

### 2.1. Comparison of Different Extraction Methods

The extraction yield of polysaccharides from *Gracilaria lemaneiformis*, which served as an example, was determined by different extraction methods (Table 1). The polysaccharide yield decreased in the following order: MAE > hot water extraction > ultrasound-assisted extraction. The MAE extracts presented the highest content of polysaccharides (9.618% ± 0.731%) while the ultrasound-assisted extracts had the lowest polysaccharide yield (8.523% ± 0.574%).

### 2.2. Optimization of Extraction Parameters and Validation of the Model

On the basis of the preliminary range of extraction variables determined from the single-factor experiment, a Box-Behnken design was used to determine optimal levels of extraction variables including liquid-to-solid ratio (*w*/*w*, A), extraction time (min, B), and extraction temperature (°C, C), for the extraction yield of polysaccharides. By applying multiple regression analysis on the experimental data, the results of regression analysis and analysis of variance (ANOVA) for the fitted quadratic polynomial models are given in Table 2, The *p*-values were used as a tool to check the significance of each coefficient, and the smaller the *p*-value, the more significant was the corresponding coefficient; significant differences were accepted when *p* ≤ 0.05. The predicted response *Y* for the yield of polysaccharides can be obtained by the following second-order polynomial equation:

*Y* = 13.85 + 0.27*A* + 0.28*B* + 0.10*C* − 0.48*AB* + 0.093*AC* − 0.17*BC* − 0.97*A*^2^ − 0.77*B*^2^ − 1.01*C*^2^ (1)

*A*, *B*, and *C* are the coded variables for water concentration, extraction time, and extraction temperature, respectively. *AB*, *AC* and *BC* are interaction coefficients among three factors.

By employing Design Expert 8.0 software (Trial Version, State-Ease Inc., Minneapolis, MN, USA) to solve the resulting second-order regression model, the optimum process parameters were obtained as follows: extraction time at 51 min, extraction temperature at 70 °C, and the ratio of water to raw material at 51 g/g. Under these optimum conditions, the extraction yield of GLP, SCP, DAP, and ULLP were 13.32% ± 0.93%, 12.49% ± 0.79%, 14.21% ± 1.03%, and 11.09% ± 0.87%, respectively.

### 2.3. Fourier Transform Infrared Spectrometer (FT-IR) Analysis

**Figure 1:**
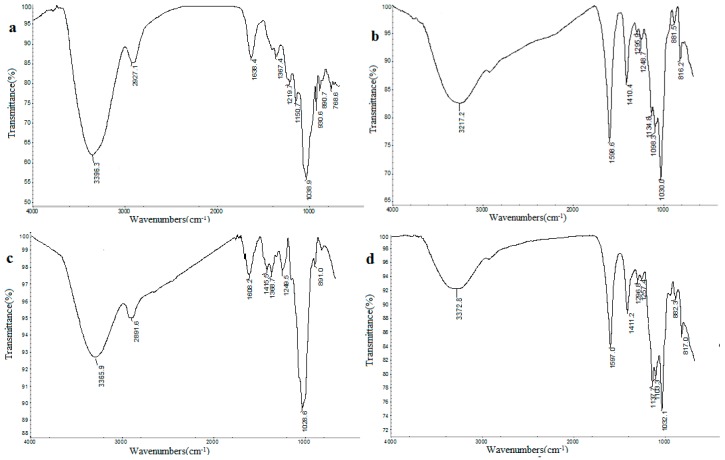
Fourier Transform Infrared Spectrometer (FT-IR) spectra of GLP (**a**); SCP (**b**); DAP (**c**) and ULLP (**d**). GLP, SCP, DAP, and ULLP are polysaccharides extracted from *G. lemaneiformis*, *S. ceylonensis*, *D. Antarctica*, and *Ulva lactuca* L., respectively.

The infrared spectra of the four seaweed polysaccharides, ranged from 400 cm^−1^ to 4000 cm^−1^, are shown in Figure 1 and Table 3. All the four samples showed typical absorption peaks of polysaccharides: the absorption bands at 3200–3400 cm^−1^, representing O–H vibrations; cm^−1^, others, in order : 2927–2891 cm^−1^ (C–H), 1638–1597 cm^−1^ (C=O), 1410–1415 cm^−1^ (C=O), 1247–1257 cm^−1^ (S=O), and 1134–1150 cm^−1^ (C–O) characteristic peaks vibrations, [8,15]; 891 and 882 cm^−1^ represent the presence of β-pyranoses [16].

### 2.4. Molecular Weight Analysis

**Figure 2:**
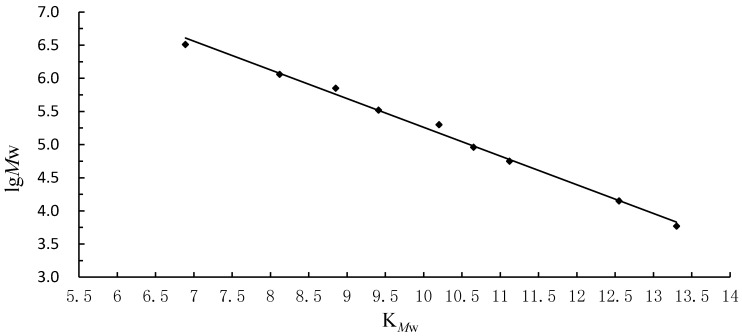
The calibration curve of molecular weight (*M*_W_) prepared from T-series dextran standards with HPGPC (high-performance gel permeation chromatography).

In a gel permeation chromatography column, the logarithm of the mean molecular weight (Lg*M*_W_) correlates with calculating elution volume (*V*_e_) from T-series standard dextrans, and each point (Figure 2) express the corresponding *K_M_*_w_ value by calculating *V*_e_ from T-series standard dextrans known molecular masses (T-890, 720, 500, 290, 196, 240, 81, 31, 11). *K_M_*_w_ is calculated using the equation:

(2) $$K_{Mw}=\frac{V_{e}-V_{0}}{V_{t}-V_{0}}$$

where *V*_e_ is the elution volume in mL, *V*_0_ for column void volume in mL, and *V*_t_ for column total volume in mL, respectively. In the present study, the average molecular weights (*M*_W_) were automatically estimated by Waters Breeze software for high-performance gel permeation chromatography (HPGPC), according to the *V*_e_ results for T-series standard dextrans, the regression equation was:

Log (*M*_W_) = −378 + 96.4 *V*_e_ + 5.75 *V*_e_^2^ + 0.19 *V*_e_^3^ (3)

with a high correlation coefficient of *R*^2^ = 0.998 obtained for the standard curve (Figure 2).

By reference to the calibration curve, the molecular weight of *SCP*, *ULLP*, *GLP*, and *DAP* were determined from their elution volumes as 404, 466, 591, and 482 kDa.

### 2.5. Monosaccharide Composition

**Figure 3:**
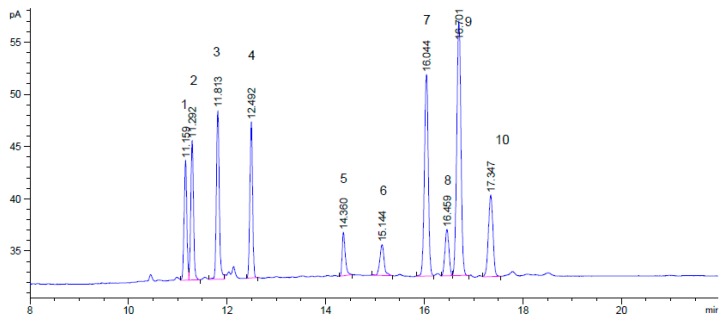
Gas chromatogram of standard monosaccharides. 1-Rhamnose; 2-Fucose; 3-Arabinose; 4-Xylose; 5 and 6-Fructose; 7-Mannose; 8-Galactose; 9-Glucose; and 10-Sorbose.

The percentages of monosaccharides in the samples were calculated from the peak areas by comparing with the nine standard monosaccharides (Figure 3). The GC–MS analysis of the acetylated derivatives of the monosaccharide from the four algal polysaccharides (SCP, ULLP, GLP, and DAP) revealed the major sugar components. The results are shown in Table 4. Glucose (Glc) widely existed in the four seaweed polysaccharides. Mannose (Man) and sorbose (Sor) presented in the seaweed polysaccharides except for GLP. Xylose occurred also in only three seaweed polysaccharides, and SCP was the one without xylose. Galactose (Gal) presented only in GLP and ULLP. Arabinose presented only in SCP and ULLP, while fucose (Fuc) presented only in DAP and ULLP. Fructose was only found in GLP. Rhamnose (Rha) was not detected in any of the SCP, ULLP, GLP, and DAP. The highest content of monosaccharides for GLP, DAP, SCP, and ULLP were galactose, glucose, mannose, and mannose, respectively. Interestingly, Fuc was found in ULLP, though it seldom occurs in polysaccharides from green seaweed. A few other green seaweed, like *Caulerpa racemosa* and *Ulva clathrate*, were also found to contain little Fuc in polysaccharide fractions [17,18]. Additionally, the content in total sugar, DAP (63.76% ± 2.12%) was much higher than in the other three—GLP (45.73% ± 1.89%), SCP (22.91% ± 1.21%), and ULLP (23.71% ± 1.29%) (Table 4).

### 2.6. Antioxidant Activities In Vitro

#### 2.6.1. ABTS Radical Scavenging Activity Assay

**Figure 4:**
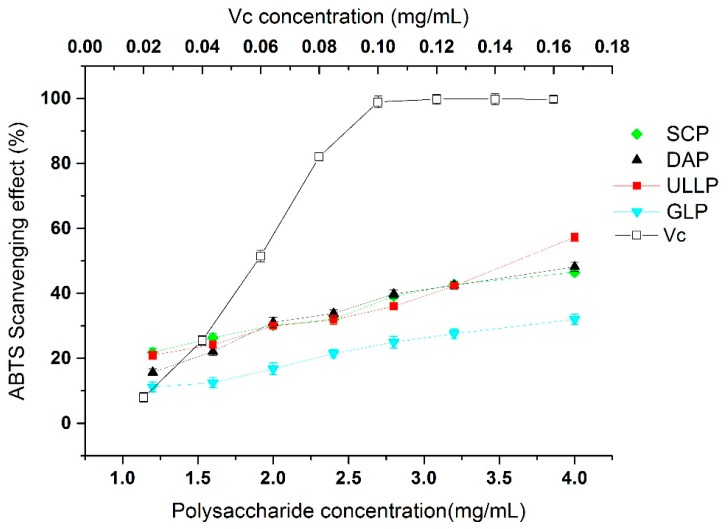
ABTS radical scavenging activities of GLP, DAP, ULLP, SCP, and Vc. GLP, DAP, SCP, and SCP are polysaccharides extracted from *G. lemaneiformis*, *D. antarctica*, *S. ceylonensis*, and *Ulva lactuca* L., respectively.

The antioxidant capacity of the sulfated seaweed polysaccharides was measured using an assay based on electron transfer; the ABTS^+^ cation was used as an oxidant. Results for the ABTS radical scavenging activity assay of GLP, DAP, ULLP, SCP, and ascorbic acid (Vc) are shown in Figure 4. Ascorbic acid was used as the reference material of anti-oxygenation. Results demonstrated that ULLP had a stronger effect on radical scavenging activity, followed by DAP and SCP; however, GLP was significantly lower than the other three polysaccharides (*p* < 0.05).

#### 2.6.2. Scavenging Activities of Hydroxyl Radical

**Figure 5:**
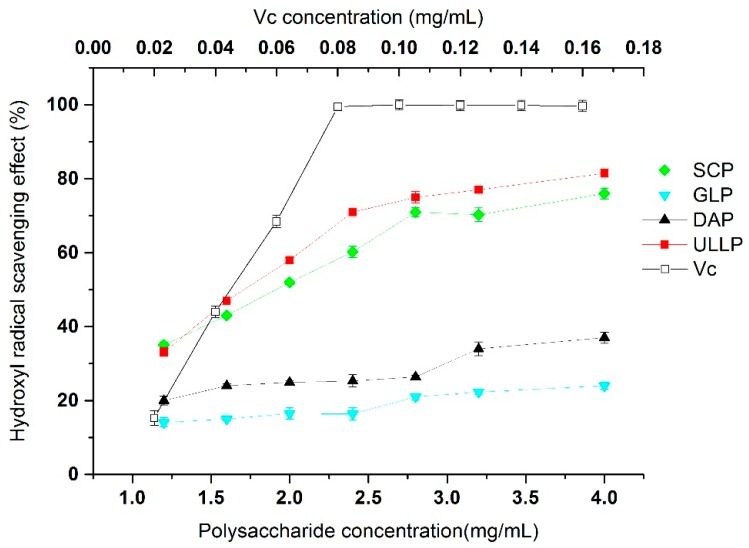
Hydroxyl radical scavenging activities of GLP, DAP, ULLP, SCP, and Vc. GLP, DAP, SCP, and SCP are polysaccharides extracted from *G. lemaneiformis*, *D. antarctica*, *S. ceylonensis*, and *Ulva lactuca* L., respectively.

Among the reactive oxygen species, hydroxyl radical is one of the most active free radical that attacks biological molecules by setting off free radical chain reactions. In this study, the hydroxyl radical scavenging activities of all the four seaweed polysaccharides and *V*c are shown in Figure 5. SCP and ULLP exhibited excellent hydroxyl radical scavenging activities, about 83.33% ± 2.31% and 80.07% ± 2.17%, respectively at 4 mg/mL, and the scavenging ability of DAP and GLP was significantly lower than that of ULLP and SCP within the range of 1.0–4.0 mg/mL (*p* < 0.05). Hydroxyl radicals are speculated to be generated from the Fe ^2+^/H_2_O_2_ Fenton reaction system [19].

#### 2.6.3. Nitrite Scavenging Activity

**Figure 6:**
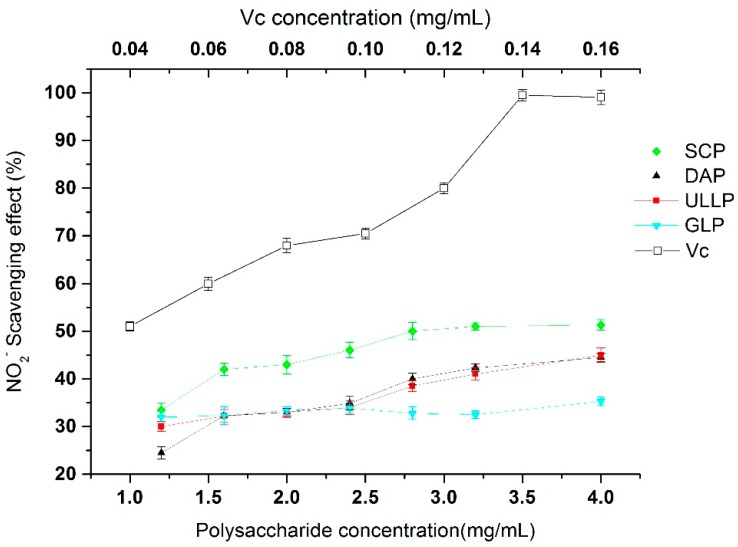
NO_2_^−^ scavenging activities of GLP, DAP, SCP, ULLP, and Vc. GLP, DAP, SCP, and SCP are polysaccharides extracted from *G. lemaneiformis*, *D. antarctica*, *S. ceylonensis*, and *Ulva lactuca* L., respectively.

Figure 6 shows the nitrite scavenging activities of GLP, DAP, ULLP, and SCP compared to *V*c. As it can be observed, the four polysaccharides samples exhibited unsatisfactory nitrite scavenging activity at all of the tested concentrations. The IC_50_ values of SLP, GLP, SMP, and DAP could not be determined within 1–4 mg/mL test dosage range.

#### 2.6.4. Determination of Reducing Power

**Figure 7:**
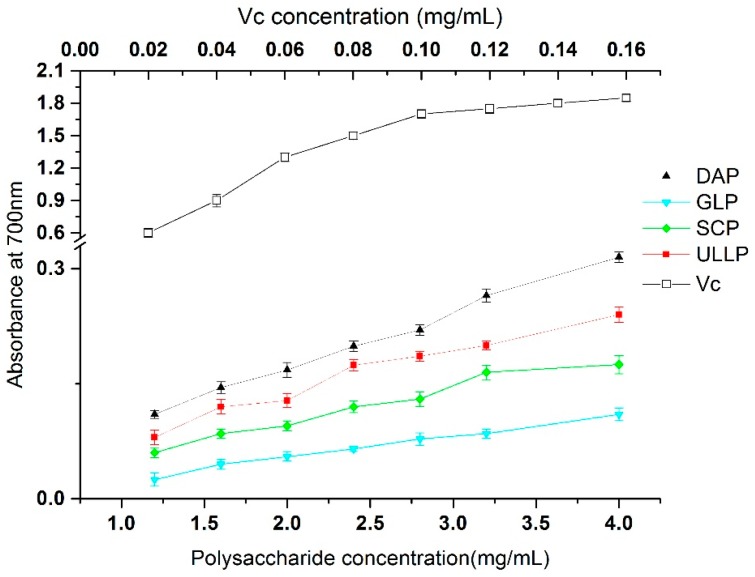
Reducing power of GLP, DAP, ULLP, SCP, and Vc. GLP, DAP, SCP, and SCP are polysaccharides extracted from *G. lemaneiformis*, *D. antarctica*, *S. ceylonensis*, and *Ulva lactuca* L., respectively.

The reducing properties were generally associated with the presence of reducing substances, which exert antioxidant action by breaking the free radical chain by donating a hydrogen atom [20]. Figure 7 reveals the reducing power of GLP, DAP, ULLP, SCP, and Vc, and the results demonstrated that DAP had the highest reducing power with a dose-dependent response at 700 nm, followed by ULLP and SCP, GLP was significantly lower than any of the other DAP, ULLP, and SCP (*p* < 0.05).

## 3. Discussion

As it can be seen in Table 1, the extraction time of GLP using MAE significantly decreased compared with the other two methods. This is because MAE offers a rapid delivery of energy to the solvent and microwave radiation can be focused directly onto the sample; thus, the heating is more efficient. Additionally, the cell walls of the seaweeds were disrupted mainly due to the mechanical effects of the microwave radiation. Similar results were already observed by Wang et al. and Wei et al. [21,22]. It was confirmed that MAE should be an appropriate and effective technique to extract polysaccharides from seaweeds because of the maximum efficiency.

The RSM of Box–Behnken design analysis results indicated that the optimized parameters for the extraction were temperature at 70 °C, time at 51 min, and liquid-to-solid ratio 51:1 (*w*/*w*). Under these conditions, the average yield of GLP (as an example) was 13.32% ± 0.93%. The effects of independent variables on the extraction yield were tested for adequacy and fitness by ANOVA, where quadratic model *p* < 0.05 was assumed to be statistically significant, and was well suited for our experiments.

The results for FT-IR spectra (Figure 1 and Table 3) showed that the strong absorbance bands around 3200–3400 cm^−1^ were due to the hydroxyl groups stretching vibrations and the small signals at 2927.1 and 2891.6 cm^−1^ of GLP and DAP indicated C–H stretching vibrations. The signals around 1600 and 1410 cm^−1^ were attributed to asymmetric and symmetric stretching of a carboxylate anion group (C=O). In addition, no absorption peak was observed at 1730 cm^−1^, characteristic signals for the deprotonated carboxylic groups. This suggests the presence of uronic acids in the four seaweed polysaccharides, as it was referred in previous reports for the uronic acids from other origins [23,24].

The peaks around 1250 cm^−1^ were caused by stretching vibrations of S–O of sulfate [5], so we might, therefore, conclude that DAP, SCP, and ULLP are sulfated polysaccharides. It has to be referred that sulfated polysaccharides, DAP and SCP, have not been reported previously. Due to bioactivities related to sulfate content, sulfated polysaccharides play an important role in regulation of the immune system, for example, stimulating immune cell proliferation and production of immune-related molecules [25,26], which might inspire more concerns in food and pharmaceutical industries. Additionally, the absorption bands between 1040 and 1020 cm^−1^ were attributed to the characteristic C–O–C glycosidic bond vibrations and ring vibrations, overlapped with stretching vibrations of side group C–O–H link bond [27]. The small absorption bands at about 898 cm^−1^ in the spectrum could be associated with β-d-glycosidic linkages between the pyranose units [8]. As the main component of DAP are glucose, the presence of β-d-glucan with pyran group in DAP could be deduced [16,28,29]. It was reported by other researchers that β-d-glucan is a polysaccharide with known significant biological functionality, for example: cytotoxic macrophage activation, anti-diabetic effects, promoting the differentiation of T cells, and so on, for some immune functions [12,30,31]. Additionally, the FT-IR spectra of ULLP and SCP are very similar, and mannose could be observed in both ULLP and SCP, with the characteristic absorptions at 880 cm^−1^, which were attributed to the β-mannose absorption peaks with a pyranose structure due to the C–H variable angle vibration of beta anomer epimers [16]. Therefore, it can be concluded that all the four seaweed polysaccharides belong to β-type polysaccharide with a pyranose group.

High performance gel permeation chromatography (HPGPC) analysis showed that the average molecular weight (*M*_W_) of GLP, DAP, SCP, and ULLP was at 591, 482, 404, and 466 kDa, respectively. The difference in the molecular weight may be caused by the differences in the degree of deacetylation and the different sources of the seaweeds [32]. Molecular weight of DAP, SCP, and ULLP are less than 500 kDa. This might be an advantage as it has been reported low-molecular-weight polysaccharides are more effective than high-molecular-weight polysaccharides at enhancing immunity [33,34], and sulfated dextrans with an average molecular weight of 10–500 kDa already showed greater anti-HIV activity [32,35]. These results are also in agreement with the ones obtained in this experiment: the three polysaccharides *(*DAP, SCP, and ULLP) proved to have more prominent antioxidant activities than GLP (*M*_W_ 5.91 kDa; not sulfated).

Comparing with the gas chromatogram of the standard monosaccharides (Figure 3 and Table 4), there were at least four monosaccharide units in every polysaccharide: GLP consisted mainly of galactose (Gal), fucose (Fru), glucose (Glc), and xylose (Xyl), with the molar ratio of 18.76:5.968:4.48:1.811; DAP consisted of glucose (Glc), mannose (Man), sorbose (Sor), fucose (Fuc), and xylose (Xyl), with the molar ratio of 26.238:2.936:2.704:1.060:0.892; *SCP* consisted of mannose (Man), glucose (Glc), sorbose (Sor), and arabinose (Ara), with the molar ratio of 14.367:5.339:2.829:1.213; and ULLP consisted of Man, Glc, Ara, Sor, Gal, and Fuc with the molar ratio of 6.659:1.931:0.519:0.461:0.277:0.222:0.194. These results suggest that the four polysaccharides were heteropolysaccharides. In addition, Gal (18.760 mole ratio) was the main sugar unit in GLP, Glc (26.238 mole ratio) was the main sugar unit in DAP, and Man (14.367 mole ratio, 6.659 mole ratio) was the main sugar unit in both ULLP and SCP, respectively, which were in agreement with the T-IR spectra.

The ABTS radical scavenging activity assay has been widely applied to evaluate antioxidant activity from the visible-light range with the maximum absorption change at 734 nm [36]. As shown in Figure 4, the ABTS radical scavenging activity of SCP, ULLP, and DAP was significantly higher than GLP (*p* < 0.05), the antioxidant activity being dose-dependent for all the SCP, ULLP, DAP, and GLP. However, the results also showed that the ABTS radical scavenging activity of ULLP was the highest at 4 mg/mL, and non-sulfated polysaccharide GLP was the lowest in the four seaweeds. Further, from Figure 4, it can be deduced that the IC_50_ values of ULLP, SCP, and DAP were 3.59 ± 0.11, 3.99 ± 0.09, and 3.75 ± 0.10 mg/mL, respectively. ABTS free radical of sulfated polysaccharide was higher when compared to that of the previous study on sulfated polysaccharide (UFP1 and UFP4) from *Ulvafasciata* seaweed which reported IC_50_ values at 5.8 mg/mL (Shao et al.) [37]. Our results also suggested that sulfate content showed a relevant effect on ABTS radical scavenging capability, which agree with previous reports from other seaweed samples [37,38]. The results would be helpful to understand the antioxidant properties of sulfated polysaccharides and further support the hypothesis that sulfate content may function as effective antioxidants. However, their bioactivity is significantly lower than that of ascorbic acid (*p* < 0.05), where IC_50_ is 0.06 mg/mL on the ABTS radical scavenging activity. The half maximal effective concentration (IC_50_) is defined as the concentration of the sample at which the scavenging rate reaches 50%, thus, a lower IC_50_ value corresponds to a stronger antioxidant activity of the sample [39].

The removal of hydroxyl radicals is important for the antioxidant defense in cell or food systems, as they attack most biological molecules by setting off free radical chain reactions, which are very harmful to the organisms [40,41]. As shown in Figure 5, the scavenging rate of hydroxyl radicals of SCP and ULLP significantly increased with the concentration (*p* < 0.05). However, GLP and DAP showed unsatisfactory hydroxyl radical scavenging activities at 1–4 mg/L tested concentrations. The IC_50_ values of SCP and ULLP were 1.81% ± 0.07% and 1.66% ± 0.09% mg/mL, respectively, indicating that *ULLP* and *SCP* had good effects on hydroxyl radical scavenging abilities. Previous reports demonstrated that some plant polysaccharides, such as *Fragrance* and *Rhizoma acori tatarinowii*, with their hydroxyl radical scavenging abilities of 81.3%, at 20 mg/mL, and IC_50_ value at 2.028 mg/mL, respectively [19,42], are significantly lower than the removal of hydroxyl radicals of SCP and ULLP. While the IC_50_ of the other fractions (GLP and DAP) cannot be determined within the test dosage range (1–4 mg/L), which indicates that DAP and GLP have lower effect than SCP and ULLP on the removal of hydroxyl radicals, this may be due to GLP and DAP molecular weights being higher than that of SCP and ULLP. This result further supports the hypothesis that low molecular weight may function as effective antioxidants, and this is in agreement with results of Zhao et al. and Zhang et al. who demonstrated that the antioxidant activities have a certain relationship with molecular weight [43,44].

Additionally, as sulfate groups have high nucleophilic characteristics they could chelate with metal ions, which would protect samples against oxidative damage [5]. In this study, all of the polysaccharides except GLP were sulfated polysaccharides, so the hydroxyl radical scavenging activity of SCP, DAP, and ULLP might be stronger than that of GLP, and this actually happened (Figure 5). Meanwhile, SCP and ULLP, which have similar FI-IR spectra, also have similar hydroxyl radical scavenging activity. Therefore, polysaccharide structure, degree of sulfation, *M*_W_, and monosaccharide composition may affect the chelating properties of polysaccharides, and also influence their antioxidant activities [25,45]. The exact mechanism underlying the hydroxyl radical scavenging activity needs to be further investigated.

Nitrite is highly toxic and the excessive presence of nitrites in the body leads to negative effects, such as the oxidation of hemoglobin, which can cause methemoglobinemia [46], and acts in many tissues to regulate a diverse range of physiological processes [47]. Therefore, the removal of nitrite from natural food is important. As shown in Figure 6, all of the samples showed nitrite scavenging effects which were correlated with a slow increase in the concentrations. The scavenging effect of the four samples increased in the following order of SCP > ULLP > DAP > GLP, with SCP presenting the highest NO_2_^−^ scavenging activity (43.97% ± 2.31%) and *GLP* being the least bioactive (35.92% ± 1.93%). In addition, the results also show that nitrite scavenging activity of sulfated polysaccharides *(*SCP, ULLP and DAP) were stronger than that of GLP. From the results it may be considered that the presence of sulfated groups in polysaccharides is also as a factor influencing nitrite scavenging activity of polysaccharides. Further, all IC_50_ values cannot be determined at 1–4 mg/mL tested concentrations, and nitrite scavenging activity with increasing tested concentrations also becomes slower and slower, the results also showing that nitrite scavenging activity of the four seaweed polysaccharides were weaker than the other antioxidant activity described above (ABTS, hydroxyl radical scavenging capacity and reducing power).

The reducing properties have been shown to exert antioxidant action by breaking the free radical chain by donating a hydrogen atom , and were reported to react with certain precursors of peroxide, thus preventing peroxide formation (and liver injury) [20,48,49]. Therefore, the reducing capacity of a compound may serve as a significant indicator of its potential antioxidant activity [15]. As shown in Figure 7, the reducing power of the seaweed polysaccharides (DAP, ULLP, SCP, and GLP), determined at 700 nm showed a dose-dependent response. DAP and GLP exhibiting the highest and the lowest reducing power, respectively. The reducing capacity of the four seaweed polysaccharides increased in the following order of DAP > SMP > SLP > GLP, with the concentrations increased to 1–4 mg/mL, their absorption values were at 0.317% ± 0.035% (DAP), 0.248% ± 0.027% (SMP), 0.176% ± 0.011% (SLP), and 0.113% ± 0.014% (GLP), respectively. Their reducing power was much weaker than that of Vc. These data suggest that the reducing power of DAP was relatively more pronounced than that of the three other polysaccharides SMP, SLP and GLP, and non-sulfated polysaccharide GLP showed the lowest reducing capacity. DAP could be an effective electron donor, capable of reacting with free radicals and converting them to more stable products [50]. Additionally, the results agree with Yuan et al. who reported a significant improvement in polysaccharide reducing power after sulfation [51]. Additionally, it was also reported that the high reducing power was more related to the phenolic acid content of seaweeds [52].

## 4. Materials and Methods

### 4.1. Materials and Chemicals

*Sarcodia ceylonensis*, *Ulva lactuca* L., *Gracilaria lemaneiformis*, and *Durvillaea antarctica* were purchased from Antarctic algae Co. (Xiamen, China), monosaccharide standards (l-rhamnose, d-glucose, d-xylose, l-fucose, d-galactose, d-mannose, l-sorbose, fructose, l-arabinose) and 2,2-azinobis-3-ethylbenzthiazoline-6-sulfonate (ABTS) were purchased from Wu Xi Sigma Chemical Co., Ltd. (Wu Xi, China). T-series dextrans were purchased from Wuhan Putus Macromolecular Sci. and Tech. Co., Ltd. (Wuhan, China). All other chemical reagents were of grade AR from Hangzhou Chemical Reagents Co. (Hangzhou, China). A103S weak anion-exchange resin was purchased from Deqing Purolite’s Co., Ltd. (De Qing, China). Ultrahydrogel^TM^ 120 and 1000 (7.8 × 300 mm) columns were purchased from Waters Co. (Milford, MA, USA). HPLC was carried out on a Waters 1525 HPLC system, 1525 HPLC pump, 2414 refractive index detector; Waters Co. (Milford, MA, USA).

### 4.2. Extraction of Polysaccharides

Four types of dried seaweeds were smashed by a grinder, and microwave-extracted with distilled water. The supernatant obtained by centrifugation (12,000 rpm, 20 min, 4 °C) was diluted with water up to 100 mL. The contents of total sugars were determined by the method of phenol-sulfuric acid. The combined supernatant was vacuum concentrated into one-tenth of the original volume and the precipitate was removed by centrifugation. Then the supernatant was mixed with six volumes of cold 95% ethanol for isolation of the polysaccharides. The precipitate was obtained by centrifugation (4000 rpm, 10 min, 4 °C) and lyophilized to obtain water soluble crude polysaccharides which were defined as *Sarcodia ceylonensis polysaccharide* (SCP), *Ulva lactuca* L. *polysaccharide* (ULLP), *Gracilaria Lemaneiformis polysaccharide* (GLP), and *Durvillaea antarctica polysaccharide* (DAP). In order to obtain a high yield of polysaccharides, different extraction methods were applied, and an extraction method selected to optimize the operating parameters with the response surface method (RSM).

Three operating parameters (extraction time, microwave power, and the ratio of solvent to sample) were investigated using the response surface method of Box–Behnken design, and batch experiments with extraction temperature (60, 70, and 80 °C), different extraction time (20, 40, 60 min), ratio of solid to water (1:20, 1:40, and 1:60 g/g) and under microwave power at 500 W, were conducted to cover all of the variables for determining the relative contribution of each one of these on the extraction of polysaccharides within Box–Behnken design. Analysis Design Expert software version 8.0 (Trial Version, State-Ease Inc., Minneapolis, MN, USA) was used for the experimental design and statistical analysis of the RSM results.

### 4.3. Purification of Polysaccharides by Radial Flow Chromatography

The above crude polysaccharides were redissolved in distilled water (*w*/*w*), and protein was removed by A103S ion-exchange chromatography in a radial flow column (Superflow-250, Sepragen Co., Hayward, CA, USA). The column was equilibrated with distilled water and eluted by a 0–0.4 mol/L linear gradient of sodium chloride at a flow rate of 30 mL/min and room temperature. The total carbohydrate content was monitored using phenol-sulfuric acid method and protein was monitored with a UV detector at 280 nm [53].

### 4.4. IR Spectra Analysis

The IR spectra of the purified structure of polysaccharides were determined using films prepared by the dried polysaccharides and KBr pellets with a 6700 Nicolet Fourier transform-infrared spectrophotometer (Madison, WI, USA), at the frequency range 400–4000 cm^−1^.

### 4.5. Molecular Weight Determination

The average molecular weight of all samples was determined by high-performance gel permeation chromatography HPGPC on a Waters HPLC system with Ultrahydrogel^TM^ 120 and 1000 (7.8 × 300 mm) columns, and a Waters 2414 RI detector, a 0.01 mol/L NaNO_3_ solution was used as mobile phase with a flow rate of 0.8 mL/min at 30 °C. Molecular mass was estimated using the linear regression that was calibrated by a T-series dextrans with known molecular masses (T-890, 720, 500, 290, 196, 81, 31, 11) as standards. A calibration curve was prepared by plotting *V*_e_/*V*_0_ (elution volume/void volume, mL) versus log molecular weight, and the molecular weight of the unknown polysaccharide was determined.

### 4.6. Analysis of Monosaccharide Composition

Monosaccharide composition analysis was determined by gas chromatograph (GC, Agilent 7890A, Agilent Technologies, Santa Clara, CA, USA) after acetylation. The polysaccharides (5 mg) were dissolved in 4 mL of 2 M trifluoroacetic acid (TFA) and hydrolyzed at 110 °C for 2 h in a sealed glass tube. The solution was evaporated to dryness and then methanol (4 mL) was added to give a clear solution, which was evaporated again to dryness. This procedure was repeated three times. After hydrolysis, the neutral monosaccharides were successively reduced with NaBH_4_ (3 mL, 2 mol/L) and acetylated with acetic anhydride (4 mL) at 100 °C for 1 h [54]. The alditol acetates (1–2 mL) were then analyzed by GC equipped with a flame ionization detector (FID) and a Agilent DB-1701 capillary column (30 m × 320 µm × 0.25 µm), the column temperature was maintained at 150 °C for 2 min, increased to 220 °C at a rate of 10 °C/min, held for 30 min. Nine standard monosaccharides were acetylated and analyzed by GC at the same conditions [55,56].

### 4.7. Antioxidant Activity

#### 4.7.1. ABTS Radical Scavenging Activity Assay

The 2,2-azino-bis(3-ethylbenzthiazoline-6-sulfonate) (ABTS) assay was employed to measure the antioxidant activity of all the four kinds of polysaccharides [57]. Radical scavenging activity was carried out by the method described by Re et al. with some modifications [58]. Briefly, ABTS radical solution was produced by mixing ABTS aqueous solution (final concentration 7 mmol/L) with potassium persulphate (final concentration 4.9 mmol/L), and the mixture was incubated in the dark at room temperature for 16 h. After incubation, the ABTS radical solution was diluted with phosphate-buffered saline (PBS, 0.01 mmol/L, pH 7.4) to obtain an absorbance of 0.70 (±0.02) at 734 nm. The polysaccharide samples were dissolved in distilled water to form the sample solutions to the final concentrations of 1.2, 1.6, 2.0, 2.4, 2.8, 3.2, and 4.0 mg/mL. The decolorization assay started by mixing the diluted ABTS solution (3.0 mL) with polysaccharides (200 μL) [7]. The mixture was allowed to react at 25 °C for 1 h and the absorbance at 734 nm was recorded as A_i_. A control sample containing the same amount of PBS and ABTS radical was measured as A_0_. The absorbance of the polysaccharides solutions and PBS with the same amount of ABTS was recorded as A_j_.

The scavenging activity on ABTS radical was calculated by the following formula:

ABTS scavenging rate (%) = [1 − (A_i_ − A_j_)/A_0_] × 100% (4)

#### 4.7.2. Hydroxyl Radical Scavenging Assay

The hydroxyl radical scavenging activity was determined according to the method reported by Gao et al. [59]. One milliliter of various concentrations of sample solutions (1.2, 1.6, 2.0, 2, 4, 2.8, 3.2, and 4.0 mg/mL) was mixed with FeSO_4_ (1 mL, 6 mM), H_2_O_2_ (0.5 mL, 6 mM) and salicylic acid–ethanol (0.5 mL, 6 mM). The reaction mixtures were incubated at 37 °C for 30 min. Then the absorbance was measured at 510 nm. Distilled water was used as the control and ascorbic acid (*V*c) was served as the positive control. The absorbance of the control group was measured as A_0_ (water instead of sample solution); A_i_ was the result for the samples and A_j_ was the absorbance for samples with water replacing the H_2_O_2_. The hydroxyl radical scavenging ratio was calculated by the following equation:

Hydroxyl radical scavenging activity (%) = [A_0_ − (A_i_ − A_j_)]/A_0_× 100% (5)

#### 4.7.3. Nitrite Scavenging Assay

A 5.0 mL polysaccharides sample solutions were mixed with sodium nitrite (1 mL, 0.01%) and citrate-phosphate buffer (pH 3.0) and adjusted to a volume of 10 mL with distilled water. The reaction solution was incubated at 37 °C for 1 h. Then 3 mL of the reaction solution were mixed with 3 mL Griess reagent. After vigorous mixing with a vortex, the mixture was placed at room temperature for 15 min and the absorbance at 544 nm was recorded as A_i_; A_0_ was the absorbance of control group (water instead of sample solutions) [60,61].

#### 4.7.4. Determination of the Reducing Power

The reducing power was assayed according to the method used by Jayaprakasha et al. [57] with some modifications. Briefly, different concentrations of polysaccharides sample solutions in water were mixed with 2.5 mL of phosphate buffer (0.2 mol/L, pH 6.6) and 1.0 mL of potassium ferricyanide (1:100, *w*/*v*) in different test tubes. The mixtures were incubated for 20 min at 50 °C. Then 1.0 mL of trichloroacetic acid (10%) and 0.5 mL FeCl_3_ (0.1%) were added to the mixture and incubated for 10 min, the absorbance was measured at 700 nm against the buffer. Ascorbic acid was used as the standard.

#### 4.7.5. Statistical Analysis

In this study, each experiment was performed three times and the results were expressed as the means of three replicate determinations ± standard deviations (SDs). The data were analyzed by an analysis of variance (*p* < 0.05). Antioxidant property results for which the *p* values were <0.05 were considered to be statistically significant. The IC_50_ was calculated by linear regression. All data were reported as the mean ± SD for three replicates.

## 5. Conclusions

In summary, under optimum reaction conditions MAE was an effective method to extract polysaccharides from seaweed samples. MAE provides higher extraction yield and can obviously reduce the extraction time. This indicates that MAE is a more environmentally friendly technique than the traditional extraction processes. Based on the single factor experiments, the RSM was used to estimate and optimize the experimental variables (extraction time, extraction temperature, and the ratio of water to raw material), and the optimum process parameters, extraction time of 51 min, extraction temperature of 70 °C, and the ratio of water to material of 51 mL/g were obtained. Under these conditions, the yields of seaweed polysaccharides for GLP, SCP, DAP, and ULLP were 13.32% ± 0.93% (GLP), 12.49% ± 0.79% (SCP), 14.21% ± 1.03% (DAP), and 11.09% ± 0.87% (ULLP), respectively. The results revealed that all the four seaweed polysaccharides belong to β-type polysaccharides with pyranose groups, and have uronic acids. The quantitative and comparative results of external standards indicated that the main monosaccharide in SCP and ULLP was mannose, GLP and DAP were mainly composed of galactose and glucose, respectively. SCP, ULLP, and DAP, are all sulfated polysaccharides. The average molecular weight (*M*_W_) of SCP, ULLP, GLP, and DAP was at 404, 404, 591, and 482 kDa, respectively. Moreover, the antioxidant activities of the four samples were investigated. The results showed that four polysaccharides exhibited antioxidant activities in a concentration-dependent manner. Among the four polysaccharides, ULLP presented the highest ABTS radical scavenging activity, but SCP and DAP also have a pronounced effect on the ABTS radical scavenging activity. SCP and ULLP exhibited excellent hydroxyl radical scavenging activity, the reducing power of DAP was relatively more pronounced than that of the three other polysaccharides. However nitrite scavenging activities of the four seaweed polysaccharides were weaker than the above described other antioxidant activities. GLP, due to the nature of its non-sulfated polysaccharide, exhibited the lowest antioxidant activity among the four seaweed polysaccharides in all tests.

## Figures and Tables

**Table 1:** Polysaccharide yield after following different extraction conditions.

Extraction Method	Hot-Water Extract	Ultrasound-Assisted Extract	Microwave-Assisted Extract
Ratio of water to raw material (*w*/*w*)	1:20	1:20	1:20
Extraction time (min)	60	60	20
Extraction temperature (°C)	70	-	70
Extraction power (W)	-	500	500
Polysaccharides yield (%)	9.143 ± 0.723	8.523 ± 0.574	9.618 ± 0.731

**Table 2:** Results of regression analysis and ANOVA for the fitted quadratic polynomial models.

Source	Sum of Squares	Degree of Freedom	Mean Square	*F*-Value	*p*-Value	Significance
Model	14.36	9	1.60	15.05	0.0009	***
*A*-ratio	0.56	1	0.56	5.32	0.0544	
*B*-time	0.64	1	0.64	6.07	0.0433	*
*C*-temperature	0.087	1	0.087	0.82	0.3960	
*AB*	0.93	1	0.93	8.73	0.0213	*
*AC*	0.035	1	0.035	0.33	0.5845	
*BC*	0.12	1	0.12	1.12	0.3251	
*A*^2^	3.99	1	3.99	37.63	0.0005	***
*B*^2^	2.47	1	2.47	23.26	0.0019	**
*C*^2^	4.29	1	4.29	40.46	0.0004	***
Residual	0.74	7	0.11			
Lack of fit	0.62	3	0.21			
Pure error	0.12	4	0.031			
Total	15.11	16				

Significant at * *p* < 0.05; Significant at ** *p* < 0.02; Significant at *** *p* < 0.001.

**Table 3:** The IR spectrum data of the four polysaccharides.

GLP ^a^	SCP ^a^	DAP ^a^	ULLP ^a^	Characteristic Peaks
**Wavenumber (cm^−1^)**				
3200–3400	3200–3400	3200–3400	3200–3400	O–H stretch vibration
2927.1	-	2891.6	-	C–H stretch vibration
1638.4	1598.6	1608.2	1597.0	Asymmetric and symmetric stretching of carboxylate anions group (C=O)
-	1410.4	1415.5	1411.2	
-	1247.8	1249.5	1257.4	Stretching vibration of S–O of sulfate
1150.7	1134.8, 1198.3	-	1137.7, 1103.3	C–O stretch vibration of secondary alcohol
1038.9	1030.0	1028.6	1032.1	The pyranose ring and glycosidic bond C–O stretching vibrations
890.7	881.5	891.0	882.3	An absorption peak at 891 cm^−1^ indicated the presence of β-d-glucan, and 880 cm^−1^ attributed to the β-mannose absorption peak, with pyran group
-	-	816.2	817.0	

^a^ GLP, SCP, DAP, and ULLP are polysaccharides extracted from *G. lemaneiformis*, *S. ceylonensisa*, *D. Antarctic*, and *Ulva lactuca* L, respectively.

**Table 4:** The monosaccharide profile, total sugar content, and *M*_W_ of the samples.

Samples ^a^	Total Sugar (%)	Neutral Sugar (Mole Ratio)									*M*_W_ (kD)
		Fuc	Glc	Gal	Ara	Rha	Xyl	Man	Sor	Fru	
GLP	45.73 ± 1.89	0	4.48	18.76	0	0	1.811	0	0	5.968	591
DAP	63.76 ± 2.12	1.060	26.238	0	0	0	0.892	2.936	2.704	0	482
SCP	22.91 ± 1.21	0	5.339	0	1.213	0	0	14.367	2.829	0	404
ULLP	23.71 ± 1.29	0.194	1.931	0.222	0.519	0	0.277	6.659	0.461	0	466

^a^ GLP, DAP, SCP, and SCP are polysaccharides extracted from *G. lemaneiformis*, *D. antarctica*, *S. ceylonensis*, and *Ulva lactuca* L., respectively.
